# Supplementary material for: High-Intensity Interval Exercise Drives Vitamin D Receptor Expression in Skeletal Muscle via Recruitment of Non-Parenchymal Cells, Not Upregulation in Muscle Fibers
Source: Nutrients. 2025 Nov 28;17(23):3733. doi: 10.3390/nu17233733 (PMC12694124; doi:10.3390/nu17233733)
Supplement: Supplementary file 1 [file nutrients-17-03733-s001.zip › nutrients-3973046-supplementary.pdf]

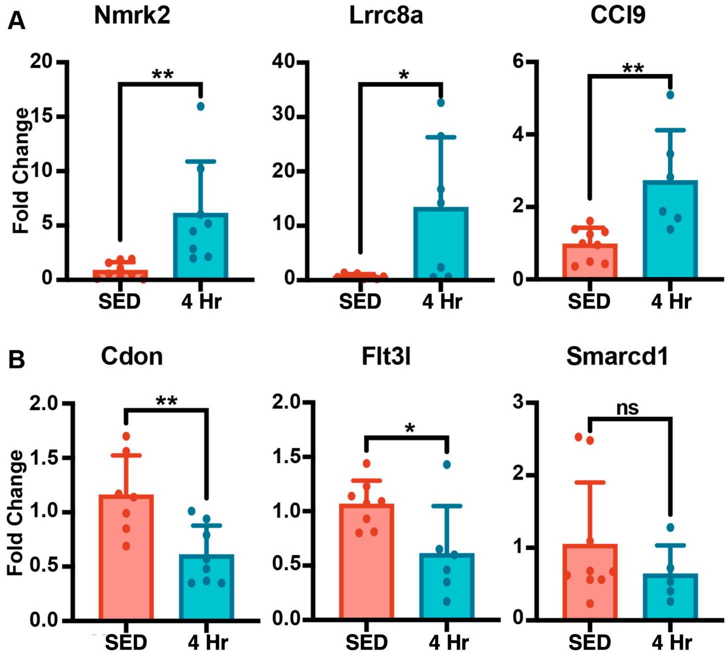

Figure S1. Validation of acute HIIE-responsive genes associated with myoblast differentiation identified by RNA-seq in the 4-h exercise group. Quantitative PCR (qPCR) was performed on gastrocnemius muscle to validate differentially expressed genes identified in the RNA-sequencing analysis described in Figure 2C. mRNA levels were measured in sedentary mice and in mice 4 h after a single bout of HIIE. Bar graphs show mean ± SD (n = 9 SED & 8, 4-h) with individual data points overlaid.

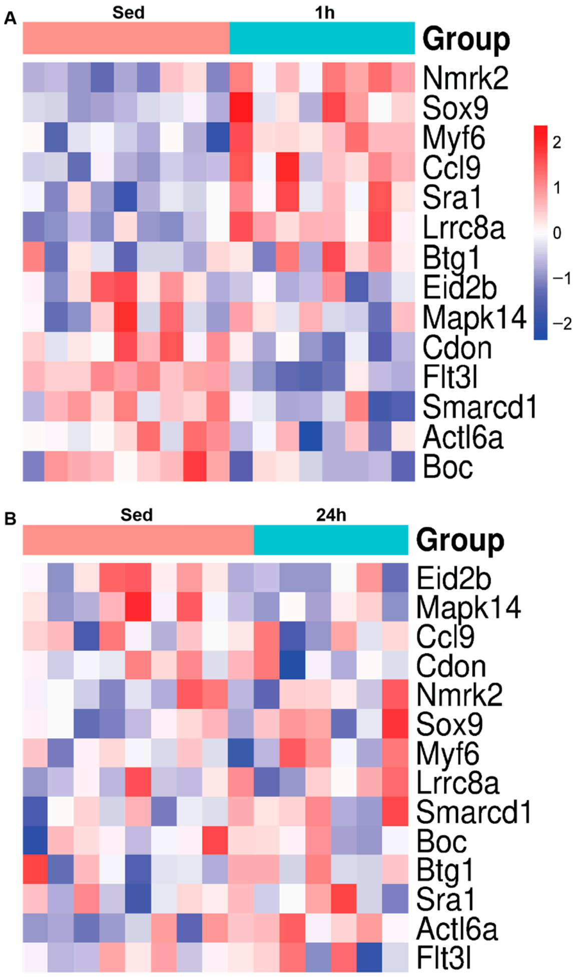

Figure S2. Differential gene expression profiles associated with myoblast differentiation by acute exercise in 1- and 24-h groups. RNA sequencing was conducted on gastrocnemius muscle tissues following acute exercise. (**A**) Heat map analysis illustrates the variably expressed genes associated with myoblast differentiation in sedentary and 1-h post-acute exercise mice, demonstrating log2-fold changes in gene expression values across each sample. (**B**) Heat map analysis illustrates the variably expressed genes associated with myoblast differentiation in sedentary and 24 h post-acute exercise mice, indicating a transient modulation of genes associated with myoblast differentiation and return to baseline after 24 h of acute HIIE. The red-to-blue color scale represents the intensity of fold changes for each gene, with red indicating an increase in expression and blue indicating a decrease. Each row represents an individual gene, while each column shows the sedentary and acute high-intensity interval training groupings.
